# Nanofibrous Conductive Sensor for Limonene: One-Step Synthesis via Electrospinning and Molecular Imprinting

**DOI:** 10.3390/nano14131123

**Published:** 2024-06-29

**Authors:** Antonella Macagnano, Fabricio Nicolas Molinari, Paolo Papa, Tiziana Mancini, Stefano Lupi, Annalisa D’Arco, Anna Rita Taddei, Simone Serrecchia, Fabrizio De Cesare

**Affiliations:** 1 Institute of Atmospheric Pollution Research (IIA)-CNR, Montelibretti, 00010 Rome, Italy; fmolinari@inti.gob.ar (F.N.M.); paolo.papa@cnr.it (P.P.); simoneserrecchia@cnr.it (S.S.); decesare@unitus.it (F.D.C.); 2 National Institute of Industrial Technology (INTI), Buenos Aires B1650WAB, Argentina; 3 Department of Physics, Sapienza University of Rome, 00185 Rome, Italy; tiziana.mancini@uniroma1.it (T.M.); annalisa.darco@uniroma1.it (A.D.); 4 High Equipment Centre, Electron Microscopy Section, University of Tuscia, University Square, Building D, 01100 Viterbo, Italy; artaddei@unitus.it; 5 Department for Innovation in Biological, Agrofood and Forest Systems (DIBAF), University of Tuscia, 01100 Viterbo, Italy

**Keywords:** molecular imprinting polymer-MIP, molecularly imprinted nanofibers-MINF, electrospinning, PVP-PAA-MWCNT composite sensor, BVOCs, terpenes, limonene selective detection, precision agriculture, environmental VOCs monitoring

## Abstract

Detecting volatile organic compounds (VOCs) emitted from different plant species and their organs can provide valuable information about plant health and environmental factors that affect them. For example, limonene emission can be a biomarker to monitor plant health and detect stress. Traditional methods for VOC detection encounter challenges, prompting the proposal of novel approaches. In this study, we proposed integrating electrospinning, molecular imprinting, and conductive nanofibers to fabricate limonene sensors. In detail, polyvinylpyrrolidone (PVP) and polyacrylic acid (PAA) served here as fiber and cavity formers, respectively, with multiwalled carbon nanotubes (MWCNT) enhancing conductivity. We developed one-step monolithic molecularly imprinted fibers, where S(−)-limonene was the target molecule, using an electrospinning technique. The functional cavities were fixed using the UV curing method, followed by a target molecule washing. This procedure enabled the creation of recognition sites for limonene within the nanofiber matrix, enhancing sensor performance and streamlining manufacturing. Humidity was crucial for sensor working, with optimal conditions at about 50% RH. The sensors rapidly responded to S(−)-limonene, reaching a plateau within 200 s. Enhancing fiber density improved sensor performance, resulting in a lower limit of detection (LOD) of 137 ppb. However, excessive fiber density decreased accessibility to active sites, thus reducing sensitivity. Remarkably, the thinnest mat on the fibrous sensors created provided the highest selectivity to limonene (Selectivity Index: 72%) compared with other VOCs, such as EtOH (used as a solvent in nanofiber development), aromatic compounds (toluene), and two other monoterpenes (α-pinene and linalool) with similar structures. These findings underscored the potential of the proposed integrated approach for selective VOC detection in applications such as precision agriculture and environmental monitoring.

## 1. Introduction

Addressing the challenge of developing sustainable agricultural practices has become increasingly imperative. With a growing global population and the widespread impacts of climate change on agriculture, adopting innovative and sustainable approaches is essential to safeguarding food security [1]. Precision resource allocation and stress mitigation strategies are crucial for sustainable productivity. The timely identification of plant stress responses is vital for yield optimization and environmental conservation. However, traditional stress detection methods are often invasive or inefficient, leading to significant shortcomings [2,3]. Advances in understanding plant defense mechanisms have led to sensor-based crop health monitoring, enhancing agroecosystem sustainability. For example, methodologies facilitating the prompt and precise detection of biotic plant stressors, such as pathogens and pests, allows for the deployment of remedial measures, to mitigate yield losses [4]. The term biogenic volatile organic compounds (BVOCs) encompasses a wide range of organic substances released by vegetation, soils, and the oceans into the atmosphere [5,6]. BVOCs are substances generally emitted by organisms for specific purposes or as a response to external or internal factors and processes [7,8,9]. Among organisms, plants typically release biogenic volatile organic compounds as a response to environmental stresses, diseases, and growth status [10,11,12]. Detecting BVOCs, like phenolics, terpenoids, alkaloids, and others, emitted by specific plant organs such as leaves, flowers, stems, and roots is an efficient yet challenging method for monitoring plant health and growth status and offers valuable insights into plant pest attacks, abiotic stressors, and additional diseases [13,14]. Their detection can be achieved through specific sensors or electronic nose-like devices [15,16,17]. The latter provides a broader perspective of detecting complex odor profiles and identifying overall patterns in BVOC emissions. However, employing multivariate statistical techniques or machine learning algorithms is necessary to discern characteristic volatile profiles. In contrast, specific sensors offer detailed information about individual BVOCs, enabling targeted analysis of crucial compounds of interest. On the other hand, developing highly selective sensors can be an equally challenging strategy. Terpenes are a class of BVOCs released by plants upon a variety of biotic stresses such as pathogenic microbes, herbivore pests, and weeds, and abiotic stresses including water availability, temperature fluctuations, light exposure, and salinity. They are also used by plants as vital signaling molecules for communicating and interacting with other organisms, from bacteria, and fungi, to insects, playing critical roles in antagonistic and mutualistic interactions, as well as in combating pest and pathogenic attacks [18,19,20,21]. Hence, a significant opportunity exists to develop selective sensors tailored to specific terpenes. For example, the emission of limonene from plants serves as a potential biomarker for monitoring plant health [15] and detecting early signs of stress [17] or disease [22]. In grapevine, limonene is particularly significant, playing a critical role during nematode attacks [23] and in responses to water stress [24]. In olive trees, limonene emissions are related to fruit ripening, and also serve as an efficient repellent against olive fruit fly (*Bactrocera oleae* (Rossi)) oviposition [13,25,26]. Therefore, the development of selective sensors for monitoring limonene could provide precise and reliable diagnostics directly linked to the agents responsible for plant stress. The proliferation of selective nanosensors for VOCs detection has seen remarkable growth alongside advancements in nanomaterials and nanotechnologies [27,28]. Molecularly imprinted polymer (MIP) technology has revolutionized the creation of polymeric matrices with tailored binding sites, precisely matching the molecular configurations of target analytes like terpenes. These MIP cavities customized for capturing analytes can operate according to a lock-and-key sensing mechanism based on size, shape, or functional group ranges and are capable of functioning for multiple cycles of detection [29]. For instance, recognition sites on MIPs accommodate analytes through hydrogen bonding, π-π bonding, and van der Waals interactions, granting sensors distinguished discrimination capabilities for the precise detection and quantification of intended compounds. The non-covalent nature of the interactions suggests that the MIP layer can repeatedly bind and release VOC molecules, allowing the sensor to function effectively over numerous uses. MIP-based sensors boast exceptional chemical and thermal stability, surpassing biomolecules, and ensuring robust performance in diverse environments [30,31]. The development of MIPs to date has involved a variety of disparate approaches, including bulk, precipitation, and emulsion polymerization, sol-gel methods, and electro-polymerization [32]. With respect to combinations of MIPs and transducers, either prefabricated MIP nanostructures could be deposited by drop casting, spin-coating, self-coating, or growing up the substrate by thermal-, photo-induced polymerization, and electro polymerization [33]. Based on transducers, MIP-based VOC sensors have been mainly exploited as chemiresistors, piezoelectric sensors, and optical sensors [34]. According to Hua et al. (2022), chemiresistors using MIPs displayed greater versatility in preparation and superior sensing capabilities compared with other MIP-based sensors for VOC detection [34]. Furthermore, higher-performance chemiresistive sensors for VOCs were achieved by combining MIPs with many conducting polymers (e.g., polyaniline, polypyrrole, and poly3-hexylthiophene), carbon-based nanomaterials (nanotubes, nanowires, nanoflakes, nanopowder), or metal/metal oxides [35,36,37]. There are several reasons why the incorporation of Molecularly Imprinted Polymers (MIPs) enhances the sensing performance of conductive polymers (CPs). The incorporation of MIPs provides highly specific binding sites for the target VOC molecules, maintaining selectivity and minimizing interference from other substances. This specificity enhances the interaction between VOCs and the sensor surface, leading to significant changes in the electrical properties of the conductive polymer, such as alterations in charge distribution or morphology. Additionally, the robust nature of MIPs contributes to the overall stability and consistent performance of the sensor under various environmental conditions. Lastly, this combination allows for a versatile sensor design, where different conductive polymers can be selected based on desired electrical properties, and MIPs can be tailored to recognize specific VOCs. According to Alizadeh et al. (2013), the combination of nanostructured materials (like MWCNTs, carbon black nanoparticles-CBNPs) increased the chance to design more effective VOCs sensors [38,39]. However, despite their excellent properties, MIP-based sensors have rarely transitioned from the academic laboratory to practical applications mainly due to several challenges in fabrication, involving costly and complex methods. Optimizing the production process to enable these products to reach consumers is paramount to facilitate efficient mass manufacturing [40]. An effort aimed at this outcome could be achieved by a combination of MIP and electrospinning (ES) nanotechnologies [41]. Indeed, electrospinning technology offers several advantages in the fabrication of chemosensors for VOCs. Firstly, this manufacturing technique is cost-effective, making it an economically viable option for the large-scale production of sensors. Additionally, electrospinning is adaptable to various deposition substrates, allowing for versatility in sensor design and integration. Furthermore, electrospinning offers an extensive selection of polymers, including eco-friendly materials, thus promoting sustainability in sensor manufacturing processes [42,43,44,45,46]. The nanoscale features of electrospun fibers enable the resulting fibrous fabrics to mimic natural structures like cilia and hair-like protrusions, pivotal in sensing the environment [47,48], and allow the creation of tunable architectures for sensor design. More specifically, in designing chemiresistors, such continuous structures can result in more efficient conduction of current signals [41]. However, such a combination presents significant challenges due to the distinct processing methodologies involved. Electrospinning embraces forming solid fibers from charged liquid jets directed towards a collector, while molecular imprinting entails selecting a template molecule, functional monomers, and a crosslinker to create specific binding sites within a polymer matrix. Overcoming differences in processing parameters and achieving compatibility between the two techniques is crucial for successful integration. Despite these challenges, ongoing research has developed innovative approaches for an effective combination [49,50,51,52]. Regarding MIP-based electrospun VOC sensors, the most investigated procedure involves fabricating and integrating MIP nanoparticles into nanofibers [53,54,55,56,57,58,59,60,61,62]. Recently, Molinari et al. (2024) reported preliminary results about the feasibility of designing a stereoselective sensor for limonene by incorporating MIPs nanoparticles (MIP NPs) (obtained by the photocrosslinking of methacrylic acid and ethylene glycol dimethacrylate monomers) into fibers of PVP containing MWCNTs [63]. The study showed that the sensor exhibited a response ~6 times higher to S(−)-limonene compared with R(+)-limonene. However, embedding MIP-NPs into electrospun nanofibers may present some challenges, including potential aggregation and uneven distribution within the polymer matrix. Moreover, non-specific interactions from the non-imprinted polymer matrix may impact recognition performance, and the incorporation of MIP particles into nanofibers can alter their physicochemical properties and subsequent recognition capacity. Conversely, an alternative approach of molecular imprinting conducted during the electrospinning process and followed by fiber mat washing might be more feasible [64]. Here, the binding sites, in the absence of a crosslinker, could be stabilized by the strong affinity between the polymer and the template, with the ability to form recognition cavities after template removal. To date, this proposed strategy is still in its infancy in sensing applications [65,66]. Macagnano et al. (2024) presented initial results for designing a stereoselective sensor for S(−)-limonene by combining MIP and electrospinning technology in a single step [67]. They employed UV-light to induce the in situ crosslinking of PVP and PAA holding S(−)-limonene as a template, and the resulting sensitivity to S(−)-limonene was 3.5 times higher than to R(+)-limonene.

The objective of the present study was to investigate and optimize the architecture of the molecularly imprinted nanofibrous sensor using a single-step electrospinning process for the selective detection of limonene. More specifically, we utilized PVP and PAA as the fiber and cavity formers, respectively, while integrating multiwalled carbon nanotubes (MWCNT) to enhance conductivity. Our approach involved the development of one-step monolithic molecularly imprinted fibers via the electrospinning technique, targeting S(−)-limonene as the molecule of interest. The functional cavities were fixed within the fibers using UV curing, followed by a thorough washing process to remove the target molecule residues. This methodology was expected to facilitate the creation of specific recognition sites tailored for limonene within the nanofiber matrix, consequently simplifying the manufacturing process. We delved into the influence of nanofiber density coverage on sensor design, as well as the effect of environmental humidity on sensing characteristics.

## 2. Materials and Methods

### 2.1. Materials

Polyvinylpyrrolidone (PVP; Mw 1,300,000, MW 30,000), Polyacrylic acid (PAA, Mw 450,000,), Multi Wall Carbon Nanotubes (MWCNT), S(−)-limonene (S-lim, 96%), α-Pinene (α-Pin, 98%), Linalool (Lin, 97%), Toluene (Tol, anhydrous, 99.8%), Hyaluronic Acid Sodium Salt from Streptococcus equi (HyA, Mw 750,000–1,000,000), Absolute Ethanol (EtOH, 99.8%), and N-N-Dimethylformamide (DMF, 99.8%) were reagent grade purchased by Merck KGaA (Darmstadt, Germany) and used without further purification.

### 2.2. Electrospinning Solutions

The electrospinning-MIP solution was prepared by blending three different mixtures. Initially, PVP was dissolved in ethanol (C = 0.12 gmL^−1^) and combined with HyA at a ratio of 1:0.031 (*w*:*w*) (Sol A). Separately, PAA was dissolved in 5 mL of EtOH at a concentration of 0.078 gmL^−1^, under magnetic stirring at 50 °C until complete dissolution (Sol B). A dispersion of MWCNT in DMF (0.2% *w*:*w*) and PVP (MW 30,000) was prepared by pulsed ultrasonication, vortexing, and magnetic stirring according to a previously described procedure [68]. Solution A, B, and the MWCNT dispersion were then mixed in proportions of 5:0.5:0.2 (*v*:*v*:*v*), respectively, under magnetic stirring until homogenization at room temperature (20 °C). Finally, S(−)-limonene was added at the final suspension as the target molecule (0.055:1, *v*:*v*). To prevent the zipper-like alignment in EtOH of polymer main chains of PAA and PVP, HyA was added to the PVP solution before complex formation [69]. The NIP solution was prepared following the same procedure as the MIP solution but without the addition of the target molecule.

### 2.3. Electrospinning Conditions and Device Fabrication

The fibers were deposited using a Fluidnatek^®^ LE-50 electrospinning machine (Bioinicia, Paterna, Valencia, Spain). To achieve regular and dry fibers, the distance between the needle and the collector was set at 12 cm, with a solution flow rate of 210 µL/h (%RH: 35, T: 20 °C). In the electrospinning setup, the needle was charged to a voltage of +5.0 kV, while the collector maintained a potential of −2 kV. A rotating drum collector (Ø: 10 cm) was employed to promote a more ordered arrangement of the fibers during deposition. Once the potential was applied, the polymeric dispersion jet coated the interdigitated electrodes (IDEs) affixed to the collector using conductive tape (500 rpm) and positioned within the deposition cone. The deposition process under the applied electrical field resulted in dry and fine fibers (NFs) collected following the jet bending and stretching processes, solvent evaporation, and subsequent splaying. Deposition times of 5, 10, and 15 min were utilized to achieve nanofibrous coverages with increasing density.

### 2.4. UV Crosslinking Process

Both MIP and NIP fibers were crosslinked using a 500 W UV lamp (Polymer Helios Italquartz, Cambiago, MI, Italy) emitting a spectrum range from 180 nm to visible) for 6 min in air, with a Peltier cell maintaining constant substrate temperature set to 28 °C. Samples were positioned 10 cm away from the light source.

### 2.5. Scanning Electronic Microscopy

The nanofibers’ size, shape, architecture, and surface characteristics were assessed using Scanning Electron Microscopy (SEM). Specifically, the electrospun nanofibrous fabrics were deposited onto thin SiO_2_ wafers, sputter-coated with gold using a Balzers MED 010 unit, and observed with a JEOL JSM 6010LA electron microscope (High Equipment Centre, University of Tuscia, Viterbo, Italy). The average fiber diameter was determined using Gwyddion© 2.64 software (GNU General Public License), with measurements conducted on 50 fibers per sample. Microsoft^®^ Excel^®^ (Microsoft 365 MSO, version N. 2402)was employed to calculate the normal probability distribution of these morphological parameters, including means and standard deviations. Measurements of fiber density coverage and porosity were obtained using the open-source software ImageJ 1.51 K (DiameterJ) (NIH, USA, W. Rasband) and were based on SEM images with dimensions of (74 × 55) μm^2^.

### 2.6. Atomic Force Microscopy

The topographies of the nanostructured layers were examined using atomic force microscopy (AFM, Nanosurf Flex-AFM, version 5—C3000, Liestal, Switzerland). Measurements were conducted in dynamic force mode (Dyn 190Al) with a resolution of 256 points per line and 256 lines scanning areas of 5 μm × 5 μm and 20 μm × 20 μm, respectively. Topography images were processed using Gwyddion© 2.64 software, with 3D visualization achieved by representing height variations with different colors, resulting in a visually informative topographical map.

### 2.7. Transmission Electron Microscope

Transmission electron microscopy (TEM) micrographs were captured at 200 keV using a transmission electron microscope equipped with an analytical double-tilt holder. Electrospun nanofibers were placed onto nickel grids (Gilder Grids, 50 mesh, 3.05 mm outer diameter, Nickel) in a static mode for a short duration and observed without fixative or staining using a JEOL 1200EXII electron microscope (JEOL, Peabody, MA, USA). Micrographs were then acquired with an SIS VELETA CCD camera (Olympus, Muenster, Germany) equipped with iTEM 2014 software.

### 2.8. Fourier-Transform Infrared Spectroscopy

Infrared (IR) spectra of nanofibers samples were collected using a Bruker (Billerica, MA, USA) Vertex 70v Michelson Fourier Transform Infrared (FT-IR) spectrometer equipped with a DLaTGS wide range detector. Spectroscopic measurements were performed in transmission mode, at room temperature (26 °C) and under vacuum conditions, in order to mitigate the interferences induced by water vapor and CO_2_ absorptions. For each IR spectroscopic measurement, 128 scans between 30 and 8000 cm^−1^ were acquired with a spectral resolution of 4 cm^−1^. The background spectrum (vacuum) was collected immediately prior to each sample measurement, with the same experimental settings of the samples. An IR spectrum of each sample resulted from the average of five repeated and independent measurements. Raw data were visualized, processed, and analyzed (absorbance calculation, baseline correction, background subtraction, cut and average, and 2nd-derivative analysis) using OPUS 8.2. software (Bruker Optics) and MATLAB (ver. 2018, MathWorks Inc., Natick, MA, USA). Background signal was subtracted calculating the IR absorption spectra A(ω) of each sample over the vacuum transmittance spectrum, then a baseline correction was applied. Second derivative analysis was performed in order to identify the positions in frequency of convoluted bands under main absorption peaks [70].

### 2.9. Electrical Measures

The interdigitated electrodes (IDEs), obtained from Micrux Technologies (Gijón, Spain), were fabricated on borosilicate substrate with IDE dimensions of 10 × 6 × 0.75 mm. These IDEs featured Pt/Ti electrodes comprising 120 pairs, each measuring 10 μm wide × 5 mm long × 150 nm thick, with a 10 μm gap between them. Before electrospinning deposition, the IDEs underwent a cleaning process involving rinsing with soap and a “base piranha” mixture (3:1, *v*:*v*, aqueous ammonia and hydrogen peroxide water solution) at 60 °C for approximately 30 min, followed by rinsing with Milli-Q water (~18 MΩ cm).

The resulting chemiresistors (IDEs + NFs) were sealed in a measuring chamber (~1 mL volume) and connected to an electrometer (Keithley 6517, Solon, OH, USA) capable of powering and measuring their electrical outputs, with data transmitted to a PC (LabVIEW 2014 Software, National Instruments, Austin, TX, USA). Clean air (5.0, Nippon gases) was used to record current under controlled humidity percentages and temperature values, applying potential values from −4.0 to +4.0 V. Resistance (R) of the fibrous coating, in the linearity range, was calculated using Ohm’s Law.

Dynamic sensor measurements were conducted at 25 °C by applying a +3 V potential between the interdigitated electrodes and using a 4-channel MKS 247 managing up to four MKS mass flow controllers (MFC), set in the range 0–200 sccm. Pure air humidified through a Nafion tube placed inside a sealed glass jar saturated with water vapors served as the gas carrier. The relative humidity was modulated by adjusting the flow rates through the tubes. Incoming air was blended with increasing concentrations of a set of terpenes (S(−)-limonene, ±linalool, ±α-pinene,) and ethanol and toluene as potential interferents. Both clean and vapor-saturated air flows converged in a mixing chamber (10 mL) before entering the measuring chamber. Each measurement was initiated after the complete recovery of the starting current (the baseline) under clean air flow. IDE responses were calculated as ΔI/I_0_, where ΔI represents current variation and I_0_ is the current under clean air flow.

## 3. Results and Discussion

### 3.1. Fibers Characterization

**Figure 1:**
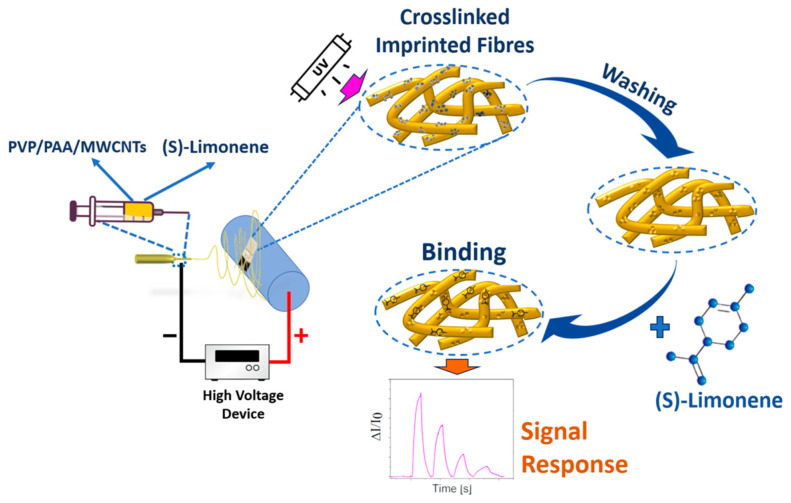
Layout of the PVP-PAA-MWCNTs sensor fabrication for S(−)-limonene detection based on the combination of electrospinning and molecular imprinting technologies via photocrosslinking and solvent washing.

**Figure 2:**
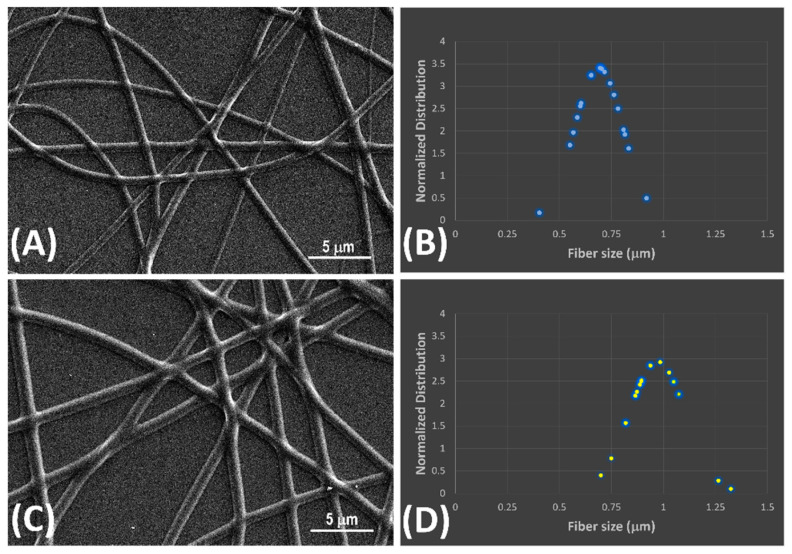
SEM images of non-imprinted (**A**) and S(−)-limonene molecularly imprinted nanofibers (**C**) collected for 5 min; Gaussian curves illustrating the size distribution of non-imprinted nanofibers (NINFs) (**B**) and molecularly imprinted nanofibers (MINFs) (**D**).

Electrospinning technology has facilitated the production of nanocomposite nanofibrous layers in a single step utilizing a single needle. A scheme of the entire deposition process and molecularly imprinted sensing fiber fabrication for detecting S(−)-limonene is depicted in Figure 1. Continuous electrospun jet streams guaranteed the formation of a fibrous network within a few minutes. UV light exposure facilitated the formation of new bonds within the single fibers (photocrosslinking), making them insoluble in ethanol (the solvent used for their fabrication), thereby acquiring novel physico-chemical properties and heightened stability [71]. The crosslinking process was verified by observing that after the 5 min electrospinning of the polymer fibers, when immersed in ethanol to remove the template (S(−)-limonene), they appeared to retain their structure without deforming or dissolving in their solvent, and remaining firmly attached to the substrates (Figure 2). 

The SEM images in Figure 2 illustrates polymer nanofibers arranged sparsely, displaying smooth surfaces and tubular forms.

These nanofibers exhibited moderate alignment, with interconnected voids that are expected to promote gas diffusion for improved efficacy in gas-VOC sensing. Aligned fibers are commonly preferred in a conductive sensor design because they provide a more direct and continuous path for electrical current to flow through the sensor. This improves the overall conductivity and reduces resistance, leading to more efficient and reproducible sensor. Figure 2B,D depicts the normalized distribution of fiber dimensions. In Figure 2C,D, molecularly imprinted nanofibers (MINFs) exhibited a narrow, well-defined diameter distribution, with a mean diameter of 977 nm and a standard deviation (SD) of ±140 nm. In contrast, non-imprinted nanofibers (NINFs) (Figure 2A,B) were approximately 42% thinner on average than MINFs (Ø: 688 ± 122 nm), with a more pronounced and upward-curving Gaussian curve shape indicating the influence of template S(−)-limonene during deposition and photocrosslinking.

**Figure 3:**
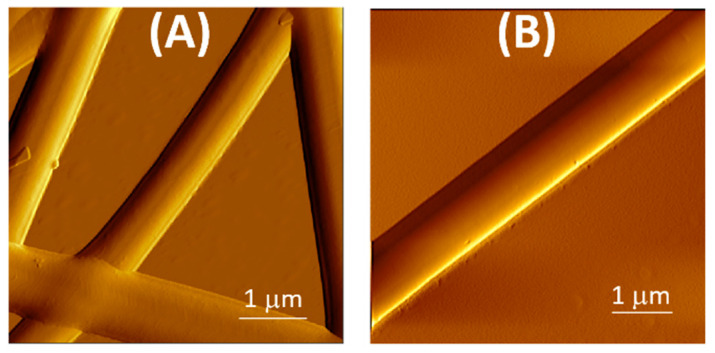
AFM micrographs of NINFs (**A**) and MINFs (**B**) (5 × 5 mm) achieved in amplitude mode.

The differences between NINF and MINF surfaces roughness were emphasized through atomic force microscopy (5 × 5 μm, achieved in amplitude mode). The AFM micrographs exhibited neither significant defects, nor irregularities on the surfaces, especially in the case of MINF (Figure 3B), where a perfectly tubular and homogeneous fiber diverged from NINFs for their slight wrinkles over the length (Figure 3A).

**Figure 4:**
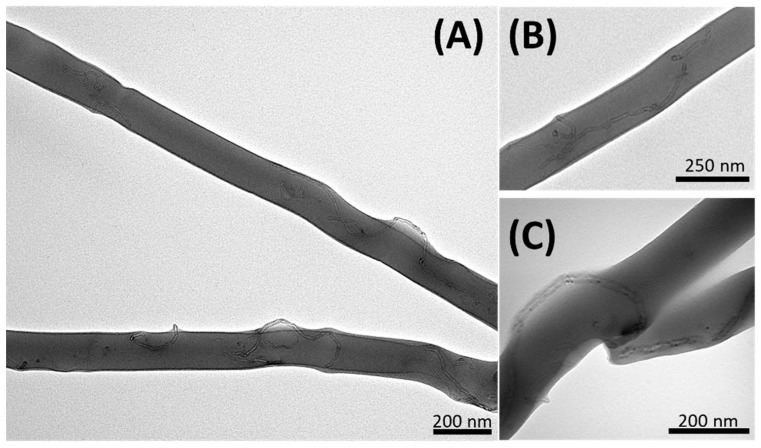
TEM images displaying nanofibers incorporating MWCNT with varying distributions: (**A**) variable distribution of MWCNTs along the MINFs, from aligned to coiled, until outward protrusions, (**B**) prevalent alignment of MWCNT inside the NINFs, as (**C**) a coiled MWCNT within a NINF encircling a cavity, and also protruding outwards, while an MWCNT is distributed longitudinally along another nanofiber.

Carbon nanotubes’ distribution and orientation inside polymer nanofibers were captured by TEM images. However, TEM micrographs provided information only about the thinnest nanofibers of MINFs and NINFs, because their size enabled the electron beam bombardment under the vacuum to provide images clear enough to display, at nanoscale resolution, the presence of MWCNT within the polymer matrix (because only thicknesses below ~300 nm are transparent to the electron beam). In Figure 4A,B, MWCNTs appear successfully embedded in the dispersing polymer matrix and well-oriented along the fiber axis, despite exhibiting some degree of tortuosity. This suggests that the original polymer dispersion contained individual nanotubes rather than aggregates or bundles. Achieving this is a common and significant challenge in developing reproducible resistive tools [66]. On the other hand, the imperfect alignment could be due to the electrospinning process, not sufficient for fully stretching the nanotubes (such as the solution viscosity, the applied electric field strength, the flow rate, the collector type, the solvent, and the interactions between the polymer blend (PVP-PAA) and the nanotubes, etc.). Additionally, intrinsic structural defects due to dislocations and vacancies can create points of irregularity in the nanotubes*’* shape and charge distribution [72]. Furthermore, in some other regions of the nanofibers, the nanotubes appeared in more irregular conformations, exhibiting bending and even protrusion out of the nanofiber (Figure 4C), mainly in conjunction with the irregularities in the nanofiber.

**Figure 5:**
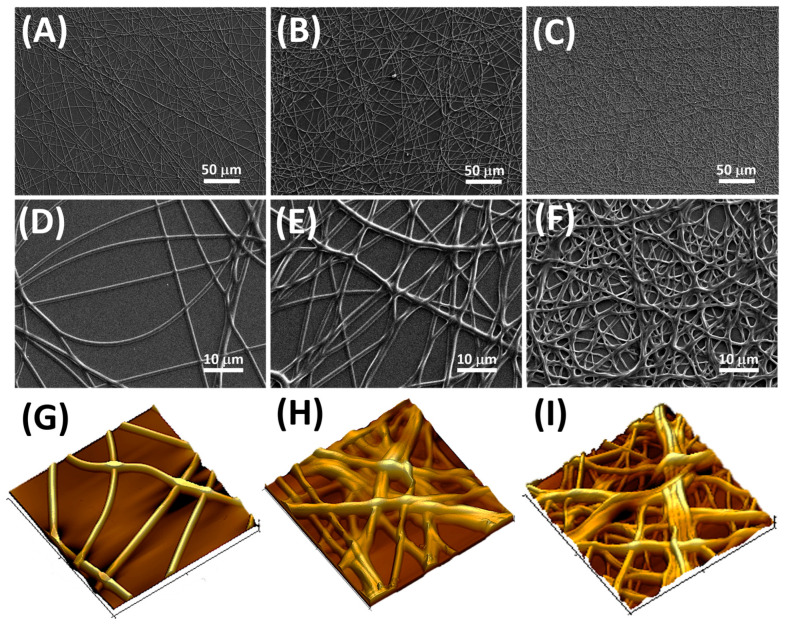
SEM micrographs depict the morphology of electrospun MINFs at various deposition durations and magnification: 5 min (**A**,**D**), 10 min (**B**,**E**), and 15 min (**C**,**F**). (**G**–**I**) Three-dimensional elaborations of AFM images for the same samples (20 × 20 μm), corresponding to increasing deposition durations, are depicted in sequence.

The SEM images in Figure 5A–F captured the evolving morphology of electrospun nanofibers (MINFs) as the deposition process proceeded. Initially, after 5 min growth, the field of view showed isolated nanofibers with relatively sparse coverage, at 39.56% (Figure 5A,D and Figure S3). These early-stage nanofibers appeared as fine threads extending across the substrate. The dipping of nanofibers in EtOH to remove the template did not appear to have influenced their distribution and shape. As the electrospinning process continued (up to 10 and 15 min, respectively), the SEM images revealed the development of a more complex and interconnected network (Figure 5B,C,E,F).

The nanofibers exhibited a denser packing with surface coverage ranging from 68% to 78%, resulting in the formation of a three-dimensional scaffold (as enhanced by AFM in Figure 5H,I and Figure S3). These fibers became entangled and overlaid, creating a mesh-like structure with increased surface area. The individual nanofibers within the network displayed trajectories intersecting, resulting in points of contact and potential bonding between adjacent fibers (Figure S3). Smaller bundles, observed in the 10 min samples (Figure 5B,E,H), consisted of a modest number of merged nanofibers, forming tight aggregations that shared a common alignment, comprising a few closely packed nanofibers. In the 15 min samples, larger bundles occurred, where a higher number of nanofibers merged together, according to a more complex architecture and a thicker and more densely packed network, and which were enhanced in the 3D elaboration of AFM images. As the deposition time increased, the porosity increased in percentage but decreased in pore size. Specifically, the pore distribution per square micron rose from 3% after 5 min of deposition to 11% and 13% after 10 and 15 min of deposition, respectively. Simultaneously, the mean pore areas were estimated to decrease from approximately 146 ± 113 μm^2^ to 87 ± 45 μm^2^ and finally to 58 ± 37 μm^2^ (Figure S3). Here bundling, branching, and surface undulations became more apparent, providing insights into the intricate architecture of the nanofiber network (Figure 5I). Ethanol washing by dipping seemed to affect the arrangement of these fibers, grouping them into bundles where individual nanofibers merged together to form cohesive structures.

### 3.2. FTIR Spectroscopy

**Figure 6:**
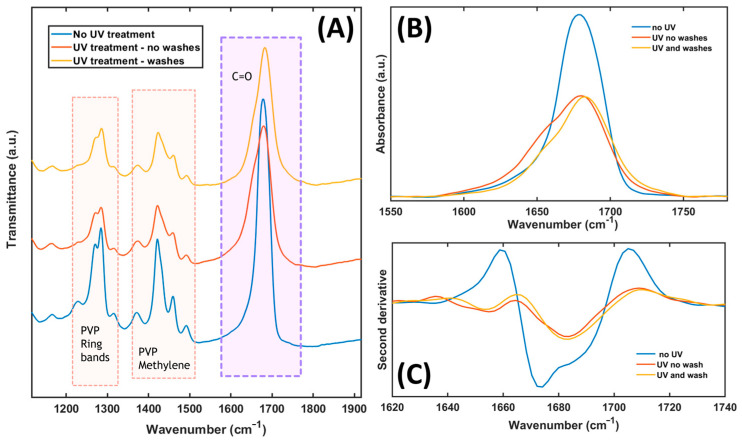
(**A**) IR absorbance spectra between 1100 and 1900 cm^−1^ of non-UV-treated nanofiber (blue) and UV-treated nanofibers without washes (orange) and with washes (yellow). (**B**) IR absorption band of C=O vibrational mode between 1550 and 1800 cm^−1^ with the same color code. (**C**) Second derivative of C=O IR absorption band between 1550 and 1800 cm^−1^ with the same color code.

FTIR spectroscopy was used to investigate the photocrosslinking effects on the nanofibers (Figure 6). As shown in Figure 6, UV-irradiated MINFs exhibited broader and less intense spectral features between 1100 and 1900 cm^−1^ compared with the non-irradiated analogous sample. This evidence indicated that UV radiation caused changes in the molecular structure of the nanofibers. In fact, in Figure 6A, a decrease in intensity could be observed in the absorption bands associated with the PVP ring (1200–1320 cm^−1^) [73] and the methylene group (1380–1480 cm^−1^). This decrease suggests that UV radiation might have induced structural changes in the polymer, through main chain scission [71]. Concerning the variations observed in the shape of the typical C=O stretching band (between 1600 and 1750 cm^−1^), they could be attributed to either interactions between PVP and PAA or to the effects of UV irradiation. This suggests changes in the chemical environment around the carbonyl groups, indicating interactions between the polymers or modifications induced by UV radiation. Figure 6B compares the C=O stretching band among the samples with and without UV irradiation and following the template elution.

The no UV-treated nanofibers and the UV-treated ones without washes both exhibited a ν(C=O) band at 1680 cm^−1^.

However, in the non-UV-treated sample, this band was narrower and more intense, indicating a higher concentration or more defined structure of the carbonyl groups [73,74]. The decrease in intensity of this band, observable in the UV-treated samples, could be linked to the photodegradation progress of the polymers, due to side group abstraction or destruction [71]. The UV-treated sample with ethanol washes showed a slightly shifted ν(C=O) band at 1684 cm^−1^. This shift may suggest changes in the chemical environment around the carbonyl groups induced by the washing process after UV treatment.

After UV treatment, a shoulder was also formed at 1653 cm^−1^, indicating the presence of additional chemical groups or structural changes induced by UV irradiation. Finally, the different shapes of the C=O bands could be attributed to interactions between PAA and PVP [71], as well as the changes induced by UV radiation [71,73,74].

The second derivative negative minima in Figure 6C shows the positions of convoluted absorption bands. There is a difference among the samples without and with UV irradiation. There is the formation of a new band around 1655 cm^−1^, a decrease in intensity of band at 1674 cm^−1^, and a shift to lower frequency of the band at higher frequencies, from 1688 to 1684 cm^−1^. These observations confirmed the nanofibers’ molecular changes induced by both UV treatment and template washing.

### 3.3. Electrical and Sensing Features

**Figure 7:**
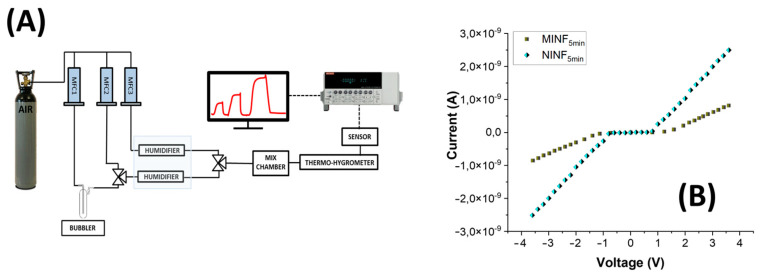
(**A**) Measuring setup includes an air cylinder, three mass flow controllers, a bubbler, and a humidifier system using Nafion^®^ tubing, a mixing chamber, the sensor within its measuring chamber, a %RH-T sensor, an electrometer, software, and a PC. (**B**) Current-voltage (I-V) curves of MINF and NINF sensors, both obtained after 5 min of deposition.

The measuring setup is depicted in Figure 7A. Figure 7B reports the current-voltage (I-V) plot for both the IDEs coated with MINFs and NINFs after 5 min of deposition, exhibiting a semiconductor behavior with a Schottky barrier. In the I-V plot, the *x*-axis represents the applied voltage across the electrode, ranging from +4 V to −4 V, with 0 V at the center. The *y*-axis corresponds to the current passing through the electrode. According to the following equation (Equation (1)):

(1) $$R=\frac{1}{2N-1}\rho \frac{w}{h\cdot L}$$

(where *N* and *L* are number and length of the fingers, *h* and *w* the electrode thickness and width, respectively, and *ρ* the resistance of the overlying material), the whole resistance is ruled by the IDE’s layout (and the resistance of the overlying material [75].

Depicted in Figure 7B, as the voltage increased positively, both MINF’s and NINF’s current remained stable until reaching a threshold at around +1 V, signalling the initiation of charge carrier injection into the nanocomposite. Beyond this threshold, a significant increase in current occurred, suggesting a Schottky barrier formation at the electrode interface, leading to semiconductor-like behavior with nonlinear characteristics. A mirroring of this trend, but with a negative sign, was observable when a negative voltage was applied. Such a symmetric contact indicates that current flows equally in both directions of voltage application within the nanofibrous layer. At higher positive or negative voltages as the potential increased, the current increased linearly for both the IDEs but with different slopes (R_NINF_: ~1.5 × 10^9^ Ohm; R_MINF_: ~2.8 × 10^9^ Ohm). The conductivity mechanism was presumably provided by a combination of charge transport within the polymer matrix generated by the conductive pathways created by MWCNTs [76]. Indeed, the addition of MWCNTs to the PVP matrix enhanced the electrical conductivity, despite PVP’s natural poor conductivity. The nitrogen heteroatom in PVP may facilitate electron acceptance, forming charge-transfer complexes. However, since the MWCNT concentration is below the percolation threshold, charge transport could likely be dominated by tunnelling. The formation of a Schottky barrier could be attributed to heterojunction at MWCNT–PVP interfaces and imperfections in the nanofiber–electrode boundary. The IV-curves were the same in shape for both MINFs and NINFs, but the resistance appeared still higher in the MINFs, probably being affected by various factors, like the density of the nanofiber network over the electrodes, the diameter of the nanofibers, and the overall morphology of the coating [77]. Indeed, NINFs arranged according to a network of thinner fibers on the electrodes, could contribute to a higher surface area per unit volume than MINFs, achieving more contact points between adjacent fibers and a larger overall interfacial area with the IDEs.

Furthermore, such an increased surface area should enhance the opportunities for charge carrier interactions and improve the probability of charge transfer between the nanofibrous layer and the electrodes. On the contrary, the MINF layer arranged into a sparser architecture may have fewer contact points and a less interconnected structure, leading to a higher resistance.

**Figure 8:**
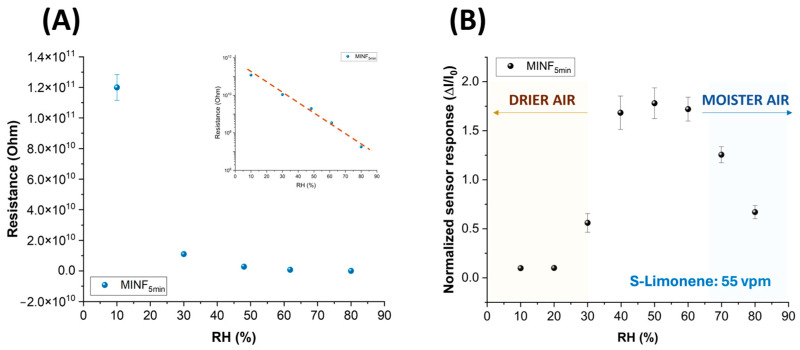
(**A**) Electrical resistance values of MINF_5min_ sensor plotted versus increasing relative humidity percentages, and (**B**) the normalized sensor responses to 55 vpm of S(−)-limonene while changing the %RH.

Figure 8A illustrates the correlation between environmental humidity levels and the conductivity of the MINF sensor. Specifically, as humidity increased, there was an exponential decrease in resistance, transitioning from approximately 10^11^ Ohms in dry air to 10^8^ Ohms in highly humid conditions. Figure 8A presents the same data on a semilogarithmic scale, facilitating an understanding of how resistance varies with differing humidity levels. The interaction mechanisms occurring at the polymeric surface of fibers with water vapors can be diverse and sometimes contradictory. The observed decrease in resistance is likely attributed to the inherently hydrophilic properties of the polymers under examination. As humidity levels rose, the polar water molecules were readily absorbed by PVP-PAA, facilitated by the layer porosity, the nitrogen atom in PVP, and the carboxylic acid groups (-COOH) along the PAA chains. These absorbed water molecules are presumed to participate in conductivity through ions, following the Grotthuss transfer mechanism [78]. Furthermore, PAA, being an acrylic acid-based polymer, could contribute to fiber conductivity through its ionizable groups. According to Pan et al. (2016) [79], ion carriers (H^+/^H_3_O^+^) are responsible for weakening the barrier at heterojunctions between polymers and MWCNTs, thus reducing the resistance in composite nanofibers and improving conductivity. However, it is worth noting that PVP and PAA are polymers known for their insulating or dielectric properties [80,81]. Therefore, in the absence of moisture and with the concentration of the MWCNT falling below the percolation threshold, the movement of charge carriers is presumably not facilitated [82,83,84]. On the other hand, when the relative humidity exceeds 60%, polymer swelling could occur, counteracting a further decrease in resistance, as depicted in Figure 8A.

**Figure 9:**
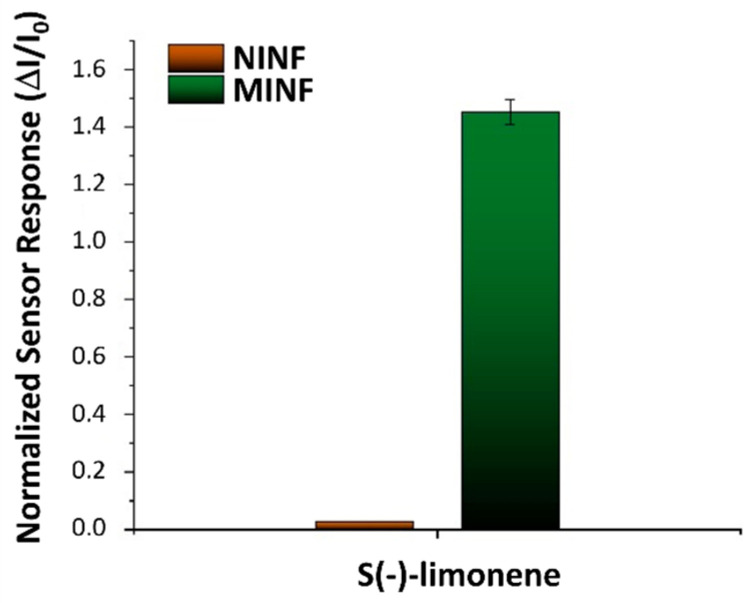
Bar plot illustrating the normalized response of NINF (Non-Imprinted Nanofiber) and MINF (Molecularly Imprinted Nanofiber) sensors to a known concentration of S-limonene (40 vpm). Measurements were calculated as the ratios between the change in current and the baseline current under clean air conditions.

The influence of humidity on the sensor features was also investigated. The MINF_5min_ sensor was exposed to a known concentration of S(−)-limonene (55 vpm) while varying the %RH. At approximately 50% relative humidity (RH), the sensor responses demonstrated the highest levels of response, stability, and reproducibility, even amidst fluctuations of %RH up to ±10% (see Figure 8B). Extreme humidity conditions (<20% RH or >60% RH) adversely affected both electrical and sensor functionalities. In lower humidity (<40%), polymer dehydration reduced free ions or charge carriers, resulting in decreased polymer conductivity and subsequent current changes when exposed to the VOC. Conversely, in higher humidity (>60% RH), polymer absorption of water molecules increased conductivity, yet sensor responses to S(−)-limonene were diminished presumably due to factors such as competition between water molecules and limonene for binding sites, and/or alterations in surface properties affecting limonene interaction. Thus, humidity control looks essential for ensuring accurate and reproducible sensor operation. Therefore, in this study, IV curves and sensing measurements were carried out at around 50% RH, representing optimal sensor operating conditions. In order to value the effectiveness of the designed sensor, NINFs and MINFs 5 min chemiresistors were exposed to an air flow containing a concentration of 40 vpm of S(−)-limonene. Both materials exhibited an increase in current; however, the response for the MINFs was approximately 55 times greater (Figure 9). The substantial disparity in responses appeared to validate the efficacy of the adopted procedure in creating “molecular cavities” within the polymer fibers, showcasing a notable affinity of MINFs for its template.

**Figure 10:**
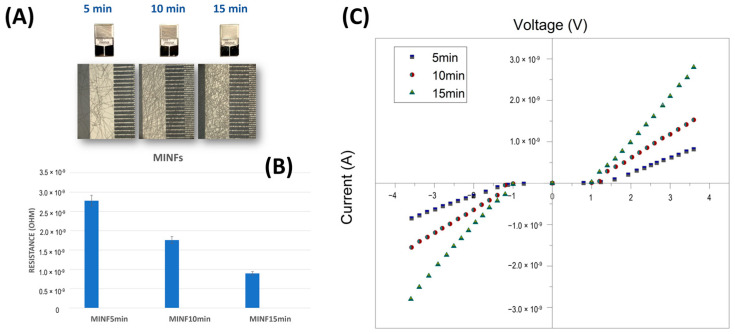
(**A**) Photographs of interdigitated electrodes after nanofiber deposition for 5, 10, and 15 min, accompanied by optical microscope magnification of a section of each of them, and a bar plot (**B**) illustrating the electrical resistance values measured for each deposition duration (R_5min_: ~2.8 × 10^9^ Ohm, R_10min_: ~1.76 × 10^9^ Ohm, R_15min_: ~8.97 × 10^9^ Ohm), and (**C**) the corresponding current–voltage curves spanning +4 to −4 Volts.

Figure 10A depicts the IDEs coated with electrospun MINFs, following the three deposition times (5, 10, and 15 min, respectively). After three different electrospinning deposition durations and template washing, the microelectrodes appeared coated with increasing fiber density. The resistance values, calculated outside the Schottky barrier region, exhibited a linear decrease with increasing deposition time (Figure 10B,C). As expected, the increase in density nanofibers over IDEs led to more conductive systems due to the increase in the number of pathways available for charge carriers to travel between the electrodes.

**Figure 11:**
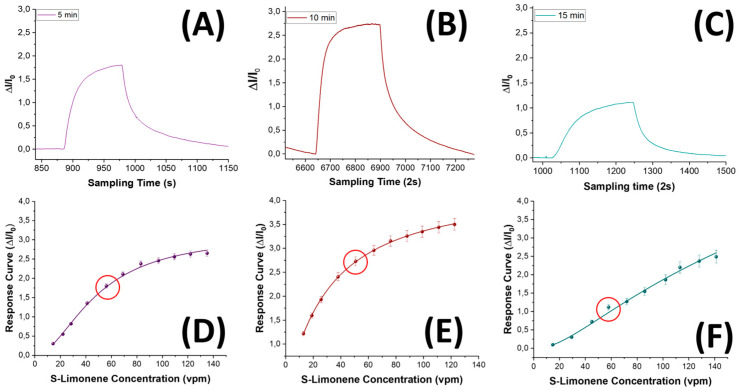
Transient responses of sensors to known concentrations of S(−)-limonene for MINF_5min_ (**A**), MINF_10min_ (**B**), and MINF_15min_ (**C**), along with the corresponding response curves (**D**–**F**). The red circles represent the concentration values related to the transient response plots.

The impact of the 5–10–15 min nanofibrous layers on S(−)-limonene detection was explored by subjecting the three sensor types to increasing concentrations of the template in air within a range spanning from 15 to 140 vpm. The sensors responses to S(−)-limonene were characterized by a swift increase in current, demonstrating rapid reactivity (Figure 11A–C). All the sensors exhibited a remarkable ability to reach a plateau within a mere 200 s according to Langmuir-like kinetics [85], indicating a prompt and effective recognition of the VOC (data reported in Table 1). Additionally, the sensors’ responses showed a consistent restoration to baseline levels when exposed to clean air, showcasing their repeatability and reliability (view Figure S2). As expected, increasing the thickness and the density of the nanofibrous layer (from 5 to 10 min electrospun sensor), the sensor responses looked improved (Figure 11B,E). Such an effect presumably was due to the increasing of the surface area of the sensing material, which allowed for a greater adsorption of S-limonene and resulted in a more pronounced sensor response.

On the other hand, a denser network of polymer nanofibers in a sensor could lead to longer response times due to diffusion limitations and intermolecular interactions. These effects appear to be substantiated by the estimated values presented in Table 1, detailing the response times (measured as t_90_, that is, the time required by the sensor to reach 90% of the response) and VOC detection limits for each sensor. As the fiber density increased, the response time exhibited an increase of up to 60%. In terms of the sensor responses, they showed a 1.5-fold enhancement when transitioning from the 5 min to the 10 min sensor, followed, conversely, by a significant decrease from the 15 min one (approximately four times smaller than the 10 min sensor response). Consequently, the limit of detection (LOD), calculated as 3 times the standard deviation of the blank, showed a decreasing trend, up to −39%, from the 5 min to the 10 min sensor. This result suggests that a denser fibrous network, although it increased the estimated response time by a few seconds, allowed for the detection of lower S(−)-limonene concentrations down to approximately 137 ppb. However, when an even denser network of fibers occurred (the 15 min one), it reversed this trend by reporting a higher value of LOD, even higher than the sensor with the poorest fiber network (more specifically about +41% and +64% than 5 min and 10 min sensors, respectively, as shown in Table 1). The latter trend could be explained by the limited accessibility of S-limonene to the active sites when fibers overlapped and merged together (Figure 5C,F), as observed in SEM and AFM images. As both the transient response shapes and the calibration curves are related to the adsorbing mechanisms resulting in the chemical affinity of the VOCs to the material, the trade-off between sensitivity and response time is a further consideration in optimizing the design of the sensor. The Langmuir-like shaped calibration curves of both the 5 min and 10 min sensors, related to the current changes when the analyte partial pressure increased, implied that adsorption occurred at specific sites on the sensor’s surface, and these sites could be occupied by only one molecule at a time. As the concentration of the analyte increased, the sensor response initially rose sharply until reaching a saturation point. At this point, the active sites on the sensor’s surface should be predominantly occupied, reaching an equilibrium between adsorption and desorption processes, and leading to a plateau in the response. This shape strongly suggests a high affinity between the sensor and the analyte. The sensor sensitivity, defined as the change in response per unit change in concentration [86], and typically calculated as the slope of the linear portion of the Langmuir-like calibration curve (*S* = Δ*I_norm_*/*C*, where Δ*I_norm_* and *C* represent the change in the current normalized to its baseline value and the analyte concentration, respectively) plays a pivotal role in determining the performance and applicability of a sensor. The calculated sensitivity for S(−)-limonene was notably higher (+25%) in the MINF_10min_ sensor than in MINF_5min_ (Table 1, Figure 11A–C,E). Conversely, the flattening of the MINF_15min_ sensor calibration curve (Figure 11F), approaching an almost linear profile with a significantly reduced slope of about 61%, described the decline in sensing performance with a continued increase in the fibrous network. This alteration may result from the slight swelling of the fibers occurring during the template elution by dipping, introducing a more tortuous quality to the fibers (Figure 5C,F). Nevertheless, this effect could also stem from a partial alteration of the molecular imprinted cavities formed along the fiber during the UV-light irradiation. Indeed, during the brief photocrosslinking procedure, it is plausible that the underlying fibers closer to the substrate may receive less UV-irradiation, potentially diminishing the effectiveness of this treatment. Alternatively, extending the exposure duration was discouraged due to the potential oxidization of limonene, as described in the Supplementary Materials paragraph and in Figure S1 [87,88] To evaluate the sensor’s ability to selectively detect its template, the MINF sensor was exposed once again to increasing concentrations of selected VOCs across the three different architectures (5, 10, 15 min). These VOCs included EtOH, used as a solvent in nanofiber development, an aromatic compound (toluene), and two different terpenes with similar molecular structures (α-pinene and linalool).

**Figure 12:**
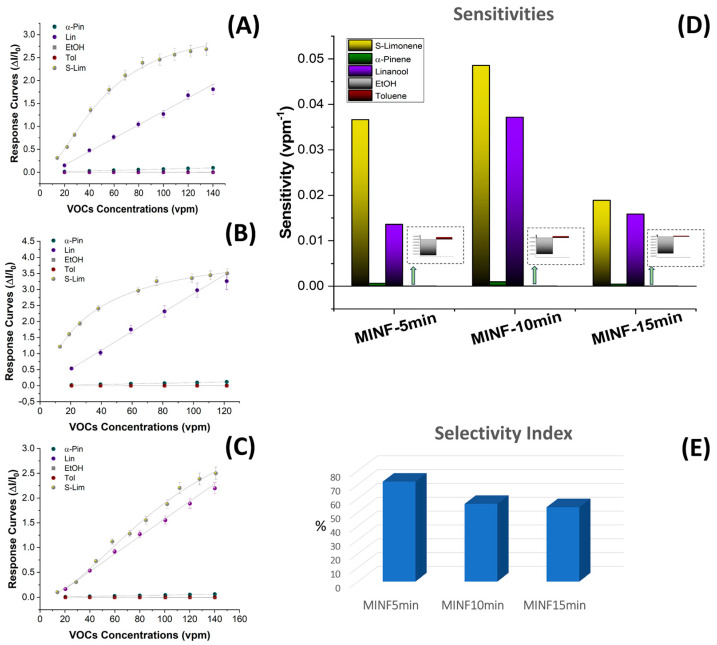
Sensor response curves of the MINFs ((**A**): 5 min; (**B**): 10 min; (**C**): 15 min) to increasing concentrations of VOCs (S-limonene, linalool, α-pinene, ethanol, toluene) ranging between 15 and 140 parts per million (vpm); (**D**) bar plot comparing sensitivity values; (**E**) 3D bar plot illustrating the selectivity index (SI).

Calibration curves for these VOCs displayed a linear shape (with the exception of limonene), indicating adsorption behavior resembling a Henry-type isotherm [89], whereas the relationship between the VOC concentration and adsorbed amount was directly proportional and not limited to join the MIP binding sites, thus indicating a lower affinity (Figure 12A). The graph in Figure 12B illustrates the estimated sensitivities of all three MINFs to these volatile compounds. While maintaining peak sensitivity and a Langmuir-like curve shape, MINF_10min_ exhibited a further reduced selectivity towards compounds with similar molecular structures, such as linalool (Figure 12B), while the other volatile organic compounds (VOCs) remained undetected. Indeed, the sensitivities to toluene and ethanol were minimal, making them imperceptible on the graph when compared with the other values (insets in Figure 12D). The minimal signal responses to EtOH, characterized by a decrease in conductivity (opposite sign in sensitivity value), appeared to result solely from a slight swelling effect. The MINF_15min_ sensor showed the poorest performance (Figure 12C,D). This outcome validates the notion that as the fiber network grows, although the number of available MIP sites increases, the washing treatment of the templates jeopardizes their sensor functionality.

Here, while confirming in all cases the greater sensitivity of MINFs to their own template (S(−)-limonene), an optimal selectivity seemed to be attained solely in the case of the thinnest fibrous network, where the sensitivity values decreased by −63% for linalool and up to −99% for the other tested VOCs (i.e., α-pinene, EtOH, and toluene).

Based on existing studies, Kikuchi et al. (2006) demonstrated the efficacy of template imprinting technology in designing a thin film sensor based on quartz crystal microbalance (QCM) for limonene detection. The study employed methacrylic acid as a functional monomer, ethylene glycol dimethacrylate (EGDMA) as a crosslinker, and 2,2′-azobisisobutyronitrile (AIBN) as an initiator. The sensor exhibited a limit of detection (LOD) of approximately 10 ppm and a selectivity of around 55%, distinguishing limonene from limonene oxide and α-pinene [90]. Ghatak et al., (2019) presented a QCM-MIP sensor prepared from the copolymer of methacrylic acid and ethylene glycol dimethacrylate for detecting R(+)-limonene in varieties of mango with a sensitivity of 0.16 Hz/ppm, repeatability and reproducibility of 98.4% and 98.8%, respectively, and with a selectivity factor of 58.16% (RH = 41%), T = 27 °C) [88,91]. Völke et al. (2022) developed a conductive molecularly imprinted polymer (cMIP) capable of detecting R(+)-limonene down to 50 ppm. Their approach involved blending polystyrene-based MIPs with poly3-hexylthiophene (P3HT) and subsequent deposition on quartz crystal microbalance (QCM) and interdigitated electrodes (IDEs) through spin- and drop-coating, respectively [92]. Their investigation revealed a relationship between template concentration and sensor response, with an initial increase in the response observed with a limonene to styrene molar ratio of 2.0:2.6. Subsequent declines in response at higher concentrations may be attributed to limonene impeding polymerization and potentially forming covalent bonds with the polymer. Following the determination of optimal proportions between the components of MIPs yielding the most favorable responses to terpenes, Iqbal et al. (2010) proceeded to construct a multiarray device. This device facilitated the monitoring of terpene emanation patterns from both fresh and dry grass, achieving a limit of detection (LOD) below 20 ppm [93]. Hawari et al. (2013) designed a membrane MIP based on methacrylic acid and a gold IDE on PET for designing a capacitive sensor for α-pinene, as a biomarker of the maturity stage of a mango. All MIP-coated IDE were then polymerized under UV light at room temperature for 6 h. The remaining molecule on the MIP can be removed by immersing it with mixture of methanol and acetic acid for the extraction of templates, thus allowing the possibility for the sensor to be used repeatedly [94].

In the present study, an estimation of sensor selectivity [92] among the tested VOCs was described by the selectivity index (SI) (Equation (2)):

SI = S_target_/∑S_interferent_ × 100 (2)

where S_target_ is the sensitivity to the defined template and S_interferents_ is the sensitivity value to the other chemicals within the measured pattern.

This parameter indicated that the MINF_5min_ sensor demonstrated a selectivity index of 72% towards the template, whereas MINF_10min_ and MINF_15min_ exhibited lower values, namely 56% and 53%, respectively.

## 4. Conclusions

When exploring the intricacies of plant communication, the monitoring of volatile organic compounds (VOCs) released under stress conditions offers a captivating glimpse into ecosystem health and vitality. Terpenes, acting as potent indicators of stressors such as drought, disease, and pollution, provide valuable insights into how plants respond to environmental challenges. By tracking these VOCs, scientists can detect stress early and devise targeted intervention strategies. This not only improves agricultural practices, leading to enhanced crop yields and resilience, but also deepens our understanding of ecosystem dynamics and the impacts of climate change. Based on the findings presented in this study, several conclusions can be drawn regarding the development and performance of molecularly imprinted nanofiber (MINF) sensors for detecting VOCs, particularly focusing on limonene as a biomarker for plant health monitoring. Our study advances the field of VOC sensing through the development and optimization of molecularly imprinted nanofiber (MINF) sensors, with a specific focus on limonene as a key biomarker for plant health monitoring. Overcoming theoretical hurdles, we innovatively address practical challenges associated with molecular imprinting during electrospinning, paving the way for the integration of electrospinning, molecular imprinting, conductive nanofibers, and rapid photopolymerization onto the transducer, successfully exploring an alternative crosslinking methods compatible with electrospinning. This comprehensive approach streamlines sensor fabrication, potentially reducing time and complexity compared with traditional methods, while enhancing sensor performance and expanding application potential in precision agriculture. MIP-based conductimetric sensors for limonene were developed using polyvinyl pyrrolidone (PVP) and polyacrylic acid (PAA) as fiber and functional cavity formers, respectively, with multiwalled carbon nanotubes (MWCNT) for conductivity. The creation of recognition sites for limonene within the nanofiber matrix holds promise for enhancing sensor performance, reducing manufacturing time, and expanding application potential in precision agriculture. Humidity control emerges as a critical factor, ensuring precise and reproducible sensor operation, with optimal conditions identified at approximately 50% relative humidity (%RH). Our sensors exhibit rapid responses to S(−)-limonene, reaching steady-state within 200 s, demonstrating their suitability for real-time monitoring applications. The impact of the thickness of the nanofibrous coating on sensor performance was investigated, revealing that increased fiber density improved sensor performance by enhancing surface area for the greater adsorption of S(−)-limonene (LOD: 137 ppb). However, excessive fiber density subjected to UV-irradiation and ethanol washing decreased the accessibility to active sites, leading to reduced sensitivity. Therefore, while all sensors exhibited great sensitivity to their target molecule, optimal selectivity (SI: 73%) is achieved with the thinnest fibrous network, highlighting the intricate interplay between sensor architecture and performance characteristics. However, in real-world applications, varying humidity levels can significantly impact sensor performance. A next step could be to integrate the device with cartridges based on Nafion^®^ membranes or tubing bathed with saline solutions at defined concentrations. This approach would help maintain the sensor’s operation within a controlled humidity range despite varying environmental conditions. Additionally, future work could focus on the development of humidity compensation algorithms or integrating humidity sensors within the system to dynamically adjust readings based on real-time humidity levels.

## Figures and Tables

**Table 1:** The sensing features of MINFs chemiresistors to S(−)-limonene.

	t_90_ (s)	LOD (ppb)	S (vpm-1)	SI (%)
**MINF_5min_**	104 ± 2	226 ± 16	36.74 × 10^−3^ ± 1.47 × 10^−3^	72.04
**MINF_10min_**	144 ± 3	137 ± 10	48.78 × 10^−3^ ± 4.08 × 10^−3^	55.99
**MINF_15min_**	270 ± 5	383 ± 27	18.98 × 10^−3^ ± 2.96 × 10^−3^	53.56

## Data Availability

All data that support the findings of this study are available after a reasonable request to the corresponding author.

## Supplementary Materials

The following supporting information can be downloaded at: https://www.mdpi.com/article/10.3390/nano14131123/s1, Figure S1: UV-Vis spectra of pure S(−)-limonene before and after UV-light irradiation; Figure S2: Transient sensors responses; Figure S3: SEM images elaboration of nanofibrous layers by Image J.
