# Supplementary material for: Nanofibrous Conductive Sensor for Limonene: One-Step Synthesis via Electrospinning and Molecular Imprinting
Source: Nanomaterials (Basel). 2024 Jun 29;14(13):1123. doi: 10.3390/nano14131123 (PMC11243275; doi:10.3390/nano14131123)
Supplement: Supplementary file 1 [file nanomaterials-14-01123-s001.zip › nanomaterials-3050260-supplementary.pdf]

Supplementary Material of the Manuscript submitted to Nanomaterials:

Nanofibrous Conductive Sensor for Limonene: One-Step Synthesis via Electrospinning and Molecular Imprinting

Antonella Macagnano, Fabricio Nicolas Molinari, Paolo Papa, Tiziana Mancini, Stefano Lupi, Annalisa D'Arco, Anna Rita Taddei, Simone Serrecchia, Fabrizio De Cesare

Materials and Methods: UV-Vis Spectrometry

The UV-2600 Shimadzu spectrophotometer (Shimadzu Italia, MI, Italy) was configured to assess the absorbance characteristics of pure limonene within a wavelength range of 200 to 800 nm. Initially sealed in opaque containers to prevent light exposure, the limonene samples were then subjected to UV-light irradiation, the same used in photocrosslinking processes (Italquarz, Italy). Quartz cuvettes containing the limonene were positioned in the path of the UV-light source and exposed for varying durations (5, 10, 20, and 40 minutes). After each exposure interval, the cuvettes were promptly removed from the UV-light source and placed into the spectrophotometer's sample compartment. Utilizing UV-Probe-2.50 Software, absorbance measurements were recorded and plotted against the corresponding exposure times, facilitating visualization of changes in limonene's absorbance profile due to UV-light exposure.

Results and Discussion

In general, limonene absorbs strongly in the UV region with peaks typically observed around 200-300 nm. These peaks are attributed to the presence of conjugated double bonds in the limonene molecule. Additionally, limonene may also exhibit absorption bands in the visible region (400-700 nm), although these are usually less prominent compared to the UV peaks.

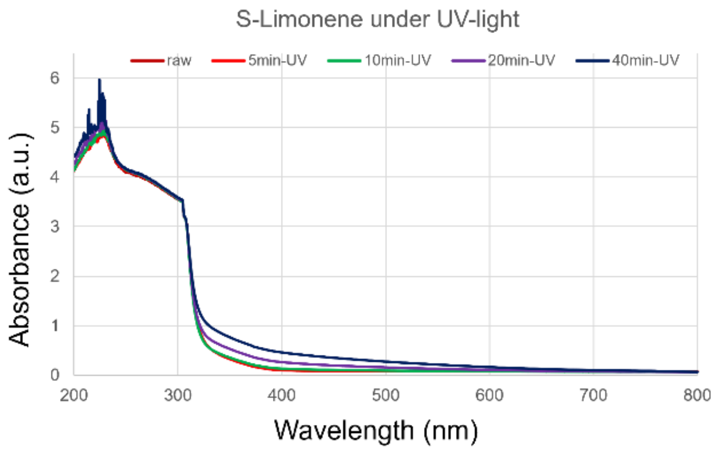

Figure S1. UV-Vis spectra of pure S(-)-limonene before and after UV-light irradiation for varying exposure durations: 5, 10, 20, and 40 minutes.

To confirm a change of the molecule following UV-irradiation, UV-Vis spectra in Figure 13 show a shift in the absorbance of S-limonene within the 300-500 nm range of the irradiated sample for more than 10 min. It could suggest ongoing oxidation processes or photolysis [**93,94**]. The specific changes in the UV-Vis spectrum would depend on the nature of the oxidized products formed during the reaction.

Indeed, the analysis aimed to discern any alterations in the spectral properties of limonene induced by UV-light irradiation, including shifts in absorbance intensity or the emergence of new spectral features.

Therefore, it is reasonable to suppose that limonene remained largely stable during the initial 10 minutes of irradiation.

This leads us to presume that there were no alterations to the template within the 5 minutes of photo-crosslinking employed for the development of the MINFs.

Transient Measurements Cycles

Dynamic sensor measurements were conducted at 25 °C by applying a +3 V potential between the interdigitated electrodes. A 4-channel MKS 247 system, managing up to four MKS mass flow controllers (MFC) set in the range of 0–200 sccm, was used. Pure air, humidified through a Nafion tube placed inside a sealed glass jar saturated with water vapor, served as the gas carrier. The relative humidity was modulated by adjusting the flow rates through the tubes. The 10-min coated sensor was subjected to decreasing concentrations of the analyte, ranging between 65 and 5 vpm, through measurement and cleaning cycles. The top graph shows the restoration of the sensor after each exposure to the VOC.
Similarly the 5-min deposited sensor was exposed to the same analyte concentration over 5 measurement cycles, demonstrating its reproducibility. More in detail, the top- graph illustrates the dynamic response of the 10min-sensor to decreasing concentrations of S-limonene, as depicted by normalized current changes over multiple measurement cycles. The x-axis represents time (sec), while the y-axis represents the normalized current changes (to the baseline current). The plot begins with a stable baseline period where only clean air is flowing through the measurement chamber, ensuring no presence of S-limonene. This period is characterized by a constant normalized current value. Following the baseline, the sensor is exposed to a specific concentration of S-limonene (65 vpm). This results in a sharp increase in the normalized current, indicating the sensor's response to the presence of the analyte. After a set duration of exposure, the flow is switched back to clean air, leading to a gradual return of the normalized current to the baseline level. This cycle of measurement and cleaning is repeated for each subsequent lower concentration of S-limonene up to 5 vpm. The plot shows multiple cycles, each representing a different, decreasing concentration of S-limonene. With each new cycle, the peak of the normalized current response decreases proportionally, reflecting the lower concentration of the analyte. The down-plot also demonstrates the reproducibility of the sensor’s response to the same concentration of S-limonene over multiple cycles, as evidenced by the consistent peak heights and

shapes for repeated exposures to 33 vpm. Overall, the plots in Figure SM2 highlight the 5min-sensor's capability to detect varying concentrations of S-limonene with a clear, measurable change in normalized current, and its ability to return to baseline after each measurement cycle, indicating good sensor stability and reproducibility.

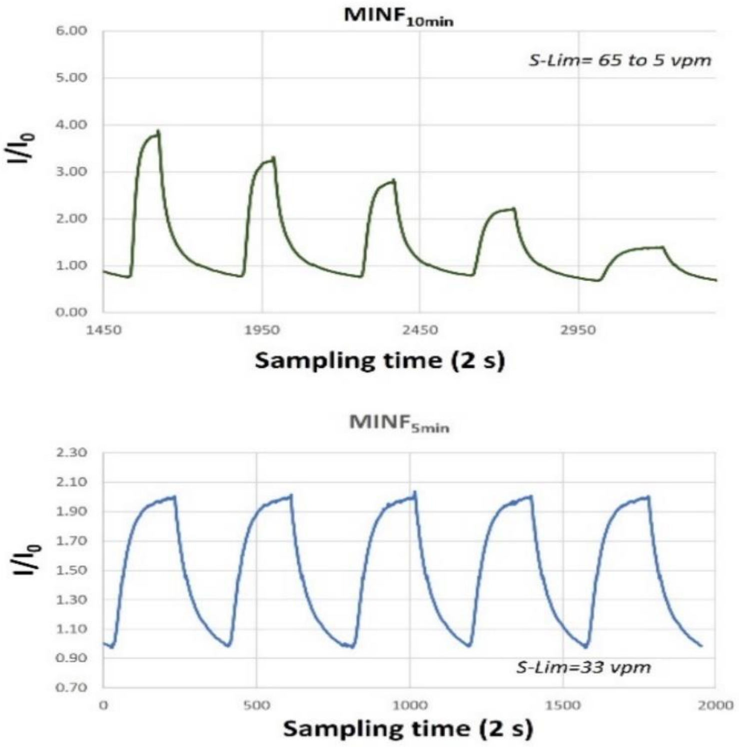

Figure S2. Transient responses of 10min-deposited sensor (top) to descreasing concentrations of S-Lim ranging between 65 and 5 vpm and of 5min-coated sensor (down) to 33 vpm (5 cycles)

SEM Image Analysis

ImageJ is a powerful, open-source image processing software developed by the National Institutes of Health (NIH). It supports a wide range of image formats and provides tools for basic and advanced image analysis. DiameterJ, a plugin for ImageJ, is specifically designed for the quantitative analysis of fiber diameters and related properties in images of fibrous materials.

In this study, ImageJ (DiameterJ) was employed to analyze and visualize the morphological characteristics of electrospun nanofibers and their changes with the increasing deposition time. More specifically ImageJ (DiameterJ) was used to identify and quantify the distribution and size of pores within the nanofiber network. This analysis involved measuring the area and frequency of pores per unit area (e.g., per square micron). The software enabled the visualization og fiber intersections, which are crucial for understanding the structural integrity and mechanical properties of the nanofiber mat. By analyzing the captured images,

ImageJ (DiameterJ) measured the diameters of individual nanofibers, as well the covered surface changes to the increasing deposition time. This data provided insights into the uniformity and consistency of the electrospinning process, as well the assumptions within the manuscript.

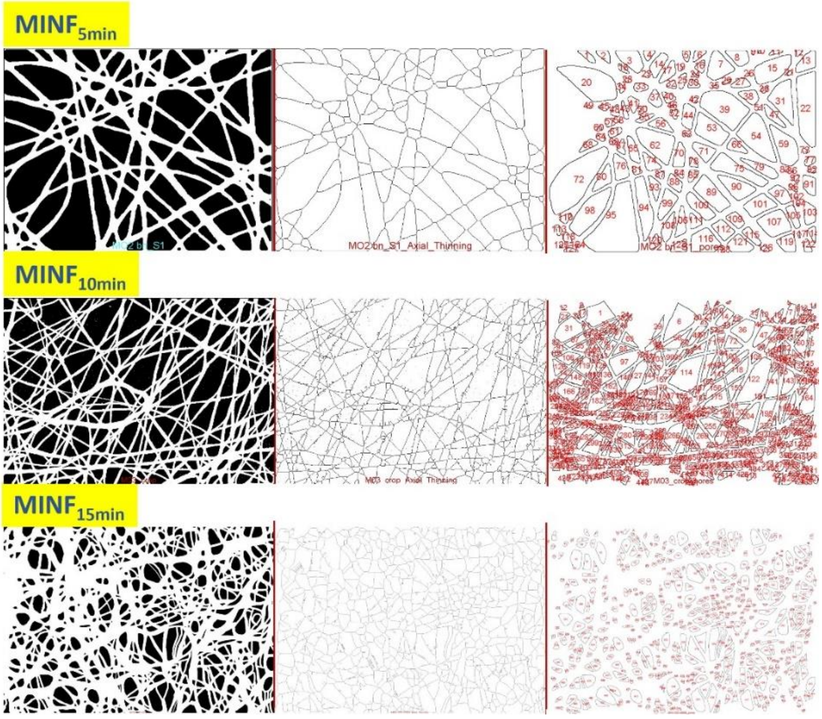

Figure S3. SEM images (74 × 55 μm) elaboration by Image J of the nanofibrous layers. In the first column, SEM images of the nanofibers are transformed into black and white, displaying the fibers at increasing deposition times from top to bottom (ranging from 5 to 15 minutes). The high contrast of these images highlights the gradual increase in surface coverage with denser nanofiber deposition. The second column schematically represents the arrangement of the nanofibers within the film, indicating potential alignment and the number of intersections between the fibers. Lastly, the third column emphasizes the distribution and size of the pores within the nanofiber network.
